# Conflict between Threat Sensitivity and Sensation Seeking in the Adolescent Brain: Role of the Hippocampus, and Neurobehavioural Plasticity Induced by Pleasurable Early Enriched Experience

**DOI:** 10.3390/brainsci11020268

**Published:** 2021-02-20

**Authors:** Alberto Fernández-Teruel

**Affiliations:** Department of Psychiatry & Forensic Medicine, Medical Psychology Unit, School of Medicine & Institute of Neurosciences, Autonomous University of Barcelona, Bellaterra, 08193 Barcelona, Spain; Albert.fernandez.teruel@uab.cat; Tel.: +34-93-5813456

**Keywords:** adolescent brain, anxiety, sensation seeking, approach–avoidance conflict, hippocampus, prefrontal cortex, amygdala, nucleus accumbens, early-life pleasurable experience

## Abstract

Adolescence is characterized both by the exacerbation of the experience of anxiety, fear or threat, on one hand, and by increased reward seeking (reward sensitivity) and risk taking on the other hand. The rise of these apparently opposite processes, i.e., threat-related anxiety and reward-related sensation seeking, seems to stem from a relatively decreased top-down inhibition of amygdala and striatal circuits by regulatory systems (e.g., prefrontal cortex, hippocampus) that mature later. The present commentary article aims to discuss recent related literature and focusses on two main issues: (i) the septo-hippocampal system (in particular the ventral hippocampus) might be a crucial region for the regulation of approach–avoidance conflict and also for the selection of the most appropriate responses during adolescence, and (ii) developmental studies involving early-life pleasurable-enriched experience (as opposed to early-life adversity) might be a useful study paradigm in order to decipher whether neuroplasticity induced by such experiences (for example, in the hippocampus and associated circuitry) may lead to better top-down inhibition and more “balanced” adolescent responses to environmental demands.

## 1. Introduction

Adolescence is a period of development characterized by an exacerbation of the experience of anxiety, fear or threat, on one hand, as well as—paradoxically—by increased reward seeking (reward sensitivity) and risk taking [1,2,3,4,5,6,7]. Related to this, it is a developmental period characterized by contrasting traits or phenotypes, such as increases in anxiety symptomatology, behaviourally characterized by avoidance, and increased risk taking behaviour (linked to reward/sensation seeking), characterized by approach responses [1]. This (apparently) paradoxical “double-edged sword”—the rise of both anxiety and sensation seeking—of adolescence seems to (at least in part) rely on the particular maturational stages and characteristics of amygdala (AMY)- and striatum-related (mainly ventral striatum, VS; nucleus accumbens, NAcc) circuits [1,2,3,4,5]. These threat- and reward-related (respectively) circuits exhibit a developmental stage during adolescence that is often characterized by increased excitability of both, whereas regulatory systems responsible for top-down inhibitory control, such as the prefrontal cortex (PFC) and the hippocampus (HPC; in particular the ventral part, vHPC), seem to mature later (e.g., [8] and references therein; see Section 2.1). These four regions interact (i.e., are interconnected) and influence the behavioural output during adolescence [1,3,4,5,8,9,10,11,12]. It is thought that when increased AMY responses to aversive (threatening) stimuli and also enhanced VS responses to appetitive (rewarding) stimuli are simultaneously present in adolescence, then the regulatory circuits/systems favour the behavioural output towards risk taking, reward seeking or approach behaviour [1,7].

In the present article, I would like to briefly discuss two main points. First, the role of the HPC (the septo-hippocampal system; see below) seems not to be just another part of the “threat” system (alongside the AMY), but it likely holds a “comparator” function which is responsible for solving conflicts, such as “threat avoidance vs. reward approach”, and selecting the most appropriate responses [9,10,11,12]. Second, I would like to make the point that in basic research (essentially with rodents), there is a bias towards studying the effects of early-life adversity or early stressful manipulations on adolescent neuroplasticity and brain development/maturation (including the HPC), whereas there is a paucity of studies addressing how positive, enriched or pleasurable early environmental experiences influence neurobehavioural processes (including the HPC) during adolescence.

In that context, early-life trauma, neglect and adversity in general are important issues to study in animals in order to better understand what their effects on the brain are and thus to be able to help humans suffering from those conditions. However, from a scientific and heuristic perspective, it is also necessary to understand what the influence of early-life positive or pleasurable (or enriched) experiences on the adolescent brain and behaviour would be. Understanding this other side of the influences of experience on the adolescent brain will put us in a better position to decipher the role of specific brain regions and circuits in adolescent psychological processes and behaviour.

## 2. Reward- and Threat-Processing Neural Circuits, and Regulatory Systems during Adolescence: A “Tetradic” Model to Account for Behavioural Outputs under Approach–Avoidance Conflict?

### 2.1. Summary of Maturational Aspects of Neural Circuits Processing Reward and Threat during Adolescence

It is far beyond the purpose of the present commentary paper to fully account for the maturational aspects of the regions and neural circuits involved in reward and threat processing during adolescence. The reader is referred to excellent recent reviews on that extremely complex issue [1,3,6,8,13,14,15,16,17]. I will just try to summarize some of the most relevant maturational and neuroplasticity aspects, and for the sake of simplicity and clarity I will emphasize the evidence related to regional and circuit function.

Dendritic spine density and type are important issues in this regard, as spines display rapid dynamic changes between immature (transient) and stable forms that are related to regional-neural function and different neuroplasticity processes [8]. In rodents, during adolescence there is a process of elimination of spines in a late-maturing region such as the PFC, whereas in a relatively early-maturing region such as the AMY there is a progressive increase in spines until adulthood [8]. The HPC is also a region whose functional connectivity (with the PFC, for example) has a more protracted maturational period than the AMY and the VS (the hubs of the threat- and reward-processing systems, respectively) [8]. There also seem to be changes in dendritic branching and spine density during adolescence in the HPC, with increases during early to middle adolescence and decreases in late adolescence and young adulthood (for review, see [15]). These findings seem to partly converge with human MRI studies showing increases in AMY and HPC volumes during adolescence, and the opposite trend for PFC [8,15], as well as with recent high-resolution MRI work demonstrating regionally heterogeneous maturation of the HPC during adolescence and extending until adulthood (see [18]).

It is relevant to highlight here that various stress experiences induce a relative spine hypertrophy in AMY, and a decrease in spine density in PFC and HPC (e.g., see reviews in [8,15]), which bears resemblance with the abovementioned adolescent dendritic spine profiles and converges with the notion of adolescence as a period of enhanced stressfulness (e.g., [1,7,8,15]).

The NAcc (ventral striatum) is interconnected with the AMY, HPC and ventromedial PFC (vmPFC). There seems to be agreement among studies showing that, like the AMY, the development of the NAcc and its connectivity mature earlier than PFC regions (e.g., [1,19]). As with the AMY, the activity of the NAcc-related mesolimbic dopaminergic system is under top-down control by the PFC and also modulated (inhibited) by the HPC (see reviews in [1,3,6,9,20]). Both the (NAcc-related) mesolimbic and mesocortical dopaminergic systems undergo developmental changes during adolescence. Among other dopamine-related changes, D1 and D2 dopamine receptor density seems to be importantly reduced during adolescence, although the issue is still controversial due to the complexity of dopamine transmission and its interactions with local VS and PFC circuits (for reviews, see [3,6,21]). In humans, a reduction of connectivity between the VS and the vmPFC from childhood to adulthood has been reported, in parallel to increased striatal volume during adolescence (see review in [6]).

At a functional level, human fMRI findings during a reward-related task suggest that the NAcc matures earlier and shows greater activity than the PFC (specifically the orbital frontal cortex [19]) in adolescents. Also, relative to adults the VS has been shown to be more activated by reward-related stimuli in adolescents, whereas during decision-making tasks activation of the PFC has shown the opposite results (i.e., PFC > VS; reviewed in [22]). These findings are in line with studies on rodents showing enhanced NAcc activity during adolescence [23]. Hence, it is conceivable that the increased activation of the VS in parallel to the decreased activation of the PFC (and thus, reduced top-down inhibitory control) may underlie risk taking and sensation seeking during that period of development [1].

On the other hand, lesions of the HPC in adult rats have been shown: (i) to increase NAcc-mediated amphetamine-induced hyperactivity and dopamine release in the NAcc; (ii) to enhance electrical brain self-stimulation; and (iii) to increase performance in positively reinforced instrumental tasks (using various reinforcers with different hedonic value—i.e., food, sucrose—under progressive ratio (PR) schedules) (see review in [24]). Remarkably, in these PR schedules the lesion group showed higher bar-pressing “break points” than the controls, thus indicating that hippocampal lesions increased the hedonic properties of rewards. All these effects of HPC lesions are likely mediated by dopaminergic mechanisms in the NAcc, which become more responsive to reward because they are “free” from hippocampal inhibitory control, in spite of an intact PFC (see review in [24]).

Hence, since the HPC and its functional connectivity are relatively late-maturing [8,18], it is conceivable that the lowered inhibitory control of the HPC over NAcc activity contributes to the predominance of reward/sensation seeking behaviour during adolescence.

For the same reason, inhibition of the threat-AMY circuitry by the HPC will also be compromised (to which decreased top-down inhibition by the PFC also contributes [6]) during that developmental period, and thus threat (stressful, anxious, fearful) experiences are more intense, and more difficult to fully extinguish, during adolescence (for reviews, see [1,8,9,10,11,12]).

To sum up, the overall regulatory (inhibitory) influence of PFC and HPC on the function of the other two regions is altered during adolescence, in such a way that AMY and NAcc are less submitted to top-down inhibitory control [6]. It is proposed that the exacerbation of anxiety-/fear- and reward-related (sensation seeking, risk taking, impulsivity) experiences and behaviours is mediated by that decreased top-down inhibitory control [1,3,8,13].

### 2.2. Shifting the Focus to “Threat–Reward” Conflict

Basic and human neurobiological research in adolescence has predominantly focused on fear, fear extinction and anxiety on one hand, and on sensation seeking and risk taking on the other (e.g., reviewed in [1,6,8,21]).

As an example of that, a recent and excellent review (in this same journal) focused mainly on whether mPFC and AMY circuits mature and are involved in fear and anxiety disorders in adolescence. Thus, Zimmermann et al. [8] exhaustively describe neural plasticity phenomena occurring in AMY, mPFC and vHPC, and the functional connectivity among these regions. The authors relate these neuroplasticity processes to fear (threat) learning, fear extinction and fear relapse, as these are phenomena that may shed light on the increased vulnerability to anxiety disorders during adolescence [8]. Other authors and reviews mainly focus on reward/sensation seeking and its circuitry during adolescence (e.g., [14]), and/or on the effects of early-life stress on either the anxiety or reward circuitry (e.g., [6,14,15]). There have been, however, valuable proposals of integrative models, such as the “triadic” model, which aims at integrating the roles of AMY, VS and PFC interacting circuitry in motivated (i.e., threat- or reward-driven) behaviour [3,13,16].

Collectively, however, the “conflict” between the two incompatible behavioural outputs derived from these opposite behavioural tendencies, i.e., threat-related avoidance and reward-related approach, has been generally underestimated in neurobiological research on adolescence. This has implications regarding how research in this field has been approached thus far (e.g., most research utilizes threat (fear)-inducing or reward-related, but not explicit conflict-inducing, procedures or tasks). Thus, with regard to threat–reward (approach–avoidance) conflict, Baker and Galvan [1] say that *“…social interaction in adolescence can be as much rewarding as it is acutely terrifying”* [1]. Likewise, Thomason and Marusak [25] state: *“Willingness to take a chance, for example, engaging a new social peer, may be frightening, but also enlivening. […] two signals compete: the potential for failure or rebuke compete with the potential for rewarding social engagement. […] One prominent theory of neurodevelopment—the triadic model […] holds that motivated behaviour is mediated by tension between reward (i.e., approach) and threat (i.e., avoidance) systems”* [25]. However, despite the fact that some authors recognize the existence of “conflict” between the two opposite response tendencies during adolescence, such a “conflict” has not been experimentally addressed from a neurobiological standpoint.

Thus, given that approach–avoidance conflict is usually present in adolescence, it is most important to address how such a conflict is solved in order to generate a given behavioural response. In this regard, an important issue is: what region/s or neural circuit/s are responsible for conflict detection and “comparing” the opposite response possibilities (e.g., threat-related avoidance response, or “approach” risk taking behaviour) and possible outcomes in order to allow adolescents to choose a given behavioural response? I propose a “tetradic” hypothesis-driven model (directly derived from J.A. Gray’s neuropsychological theory [10,11,12,26,27,28]), which, alongside the other three structures (AMY, PFC, NAcc), includes the HPC as the conflict detector and “comparator”.

## 3. Approach–Avoidance Conflict in Adolescence: The HPC as a Comparator and Regulator within the “Tetradic” Model

**Figure 1:**
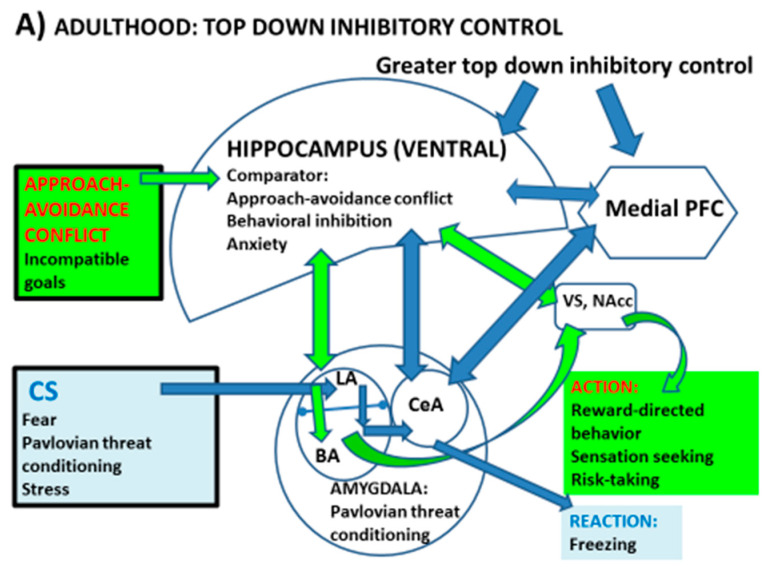

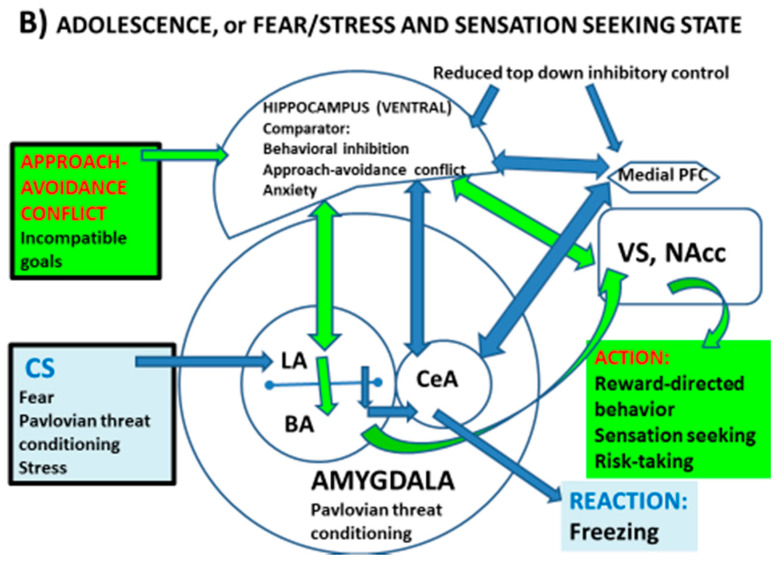
Proposed “tetradic” model of interactions and connectivity among fear/threat (AMY)- and reward (VS)-related circuitry and the degree (indicated by the size of the structures) of top-down inhibitory control by the vHPC and mPFC on AMY and VS (NAcc), which seems to be optimal during adulthood (**A**) and decreased during adolescence (**B**), one of the likely reasons being that the vHPC and mPFC (and at least part of their related circuitry/connectivity) mature later than the other two regions. This would imply that during adolescence (**B**), both threat reactions and reward-linked processes or actions would be simultaneously facilitated. CS; aversive conditioned stimulus. LA; lateral amygdala; BA; basal amygdala. CeA; central amygdala. Other symbols as in the text. Based on Fernandez-Teruel and Tobeña, 2020 [9] and references therein.

A large body of evidence, accumulated since the 1970s, supports the notion of a septo-hippocampal system acting as a comparator device and monitor of potential outcomes for approach–avoidance conflicts to select the appropriate responses [9,10,11,12,26,27,28,29]. According to J.A. Gray’s model, the septo-hippocampal (SHPC) system is interfaced between the threat (avoidance)- and reward (approach)-processing systems, and is thought to be the neuroanatomical basis of anxiety and behavioural inhibition (Figure 1; see review in [9]). The probability of approach responses in the face of approach–avoidance conflict diminishes with (is negatively associated with) the activity of the SHPC system [9,10,11,12,26,27,28,29]. Accordingly, SHPC lesions, particularly vHPC lesions (e.g., [12,28]), facilitate approach–avoidance conflict resolution by increasing active (approach) responses or decreasing passive avoidance in the face of potential threat or frustrative non-reward or reward down-shift [9,10,11,12,29,30]. Anxiolytic drugs, which disrupt theta rhythm (known to be linked to anxiety) in the SHPC, facilitate approach behaviour under these conflict conditions [9,10,11]. The SHPC system appears to act as a conflict detector and comparator under conditions of competing goals (e.g., approach–avoidance conflict) to select an optimal response (Figure 1a). In sum, septo-hippocampal lesions (and more specifically, vHPC lesions; as well as anxiolytics) facilitate approach responses to potential threat, which implies the reduction of threat- or conflict-induced behavioural suppression, whereas facilitation of the SHPC theta rhythm is associated with behavioural inhibition (e.g., freezing, passive avoidance responses) and anxiety [9,10,11,12,26,27,28,29,30] (Figure 1a).

Crucial evidence in support of the SHPC system as a comparator of (conflict-related) competing goals/responses is that septo-hippocampal (and vHPC) lesions lead to approach/active responses under most approach–avoidance conflicts in rats, both unconditioned and conditioned, and such a pattern of effects is not reproduced by AMY, VS or PFC lesions (reviewed in [9,10,12,29,30,31,32]).

Importantly, recent human studies give crucial support to the role of the HPC in solving approach–avoidance conflicts. Bach et al. [33] tested healthy participants in a novel computerized approach–avoidance task involving different threat levels. They showed increased behavioural inhibition/avoidance responses and activity of the anterior hippocampus as the probability of threat (approach–avoidance conflict intensity) increased. The authors also evaluated patients with temporal lobe epilepsy (with hippocampal sclerosis) in the same task. These patients showed behavioural inhibition (passive avoidance) deficits across the different levels of conflict in the task. This study evidenced for the first time in humans a causal relationship between the function of the anterior hippocampus and behavioural inhibition under conditions of approach–avoidance conflict [33]. Consistently, the notion of a hippocampal system acting as a threat monitor device and comparator under conditions of approach–avoidance conflict has been replicated and extended in other outstanding recent studies [30,34,35,36,37,38].

As said earlier, adolescence seems to be characterized by enhanced sensation/reward seeking (i.e., approach responses, risk taking) as well as by increased anxiety and vulnerability to anxiety disorders in parallel (or related) to deficits in fear extinction. These behavioural patterns are likely related to deficits in top-down inhibition, which lead to excessive activity of both the fear (AMY)- and reward (VS)-processing circuitry (e.g., [1,8]) (Figure 1b). As clearly stated by Zimmermann et al. [8], evidence seems to be very consistent regarding basolateral amygdala (BLA) hyperactivity and blunted mPFC inhibition of BLA during adolescence, a period in which the functional connectivity of the vHPC with BLA and both the prelimbic and infralimbic aspects of the mPFC also seems to be altered (i.e., immature), contributing to the anomalies in fear learning and extinction observed in this developmental phase [8] (Figure 1b).

However, as said above, during adolescence the hyperactivity of the fear (AMY)-related circuitry is in “tension” with the simultaneous increased activity of the reward (VS)-processing network, with the result of this “conflict” being biased towards a predominance of risk taking or reward (sensation) seeking behaviours. A point that I would like to make here is that while AMY functional interactions with mPFC and vHPC have been the focus of many studies (e.g., [8]), there is comparatively much less research devoted to HPC→ VS modulation during adolescence, even though such an interaction may have a very relevant role in conflict solving during adolescence and may help to explain the predominant behavioural profiles observed during this period of life.

In this context, studies in adult rats show that the activity of the VS is regulated by the vHPC (Figure 1). As said in Section 2.1, disruption of activity of the vHPC leads to hyperactivity of the mesolimbic (including the NAcc (VS)) dopaminergic system (e.g., [20,39], and see review in [24]). Given the late maturation of the HPC and its connectivity, these findings suggest that impaired inhibitory modulation of the VS by the vHPC during adolescence might be a relevant factor to explain the predominant sensation/reward seeking behavioural patterns in this period of life (Figure 1b). Consistently, we and others have shown that genetically selected rats displaying impulsivity and high sensation/drug/reward seeking behavioural profiles, such as the Roman high-avoidance (RHA) rat strain, present a reduced HPC function and volume, which is paralleled by enhanced NAcc activation by abused drugs and natural rewards [40,41]. Most interestingly, this phenotypic profile of adult RHA rats is paralleled by an increased density of immature pyramidal dendritic spines in the PFC, and by a pattern of pre- and postsynaptic PFC and HPC markers that are reminiscent of an immature adolescent brain [41,42,43]. That is to say, adult rats that show a predominance of impulsivity and approach (i.e., risk taking, reward-directed, sensation seeking) behaviours, and relatively decreased threat-related responses, display an adolescent-like pattern of HPC and PFC function [41,42,44,45].

## 4. How Does Early-Life Pleasurable-Enriched Experience Influence Threat and Reward Circuitry?

While a considerable amount of basic research has been devoted to ascertaining the effects of early-life adversity (e.g., different types of early-life stress or trauma) on adolescent brain function, there is a paucity of studies focused on how infantile-juvenile pleasurable experiences shape the adolescent brain and behaviour. It is beyond the scope of this paper to revise these studies on the effects of early-life adversity on the adolescent brain and behaviour, and the reader is referred to excellent recent reviews (e.g., [8,14,15,46,47]).

Neonatal handling (H; usually administered during the first 2–3 weeks of life) and juvenile-adolescent environmental enrichment (EE; usually administered for several weeks, from puberty to early adulthood) in rats have been shown to increase signs of positive affect, as measured, for example, by (hedonic) 50-kHz ultrasonic vocalizations, saccharin preference and social play behaviour [48,49,50,51,52]. These findings are compatible with the notion that H and EE treatments constitute *pleasurable* infantile-juvenile experiences for rats. It is important to highlight here that social play behaviour in rats has also been related to the function of the SHPC system ([53]; see review in [48]).

Both H and EE treatments have been reported to long-lastingly reduce fearfulness, anxiety and vulnerability to stress, as well as to improve cognition in laboratory rodents [54,55,56,57]. Furthermore, both manipulations lead to lifelong protective effects against age-related cognitive deficits and hippocampal neurodegeneration [54,55,56,58].

In addition, in adult rats H improves attention-related function, as shown by improved latent inhibition and prepulse inhibition, although these effects depend upon sex and rat strain [57,59]. Importantly, H improves working memory, and reduces anxiety and the stress-induced hypothalamic–pituitary–adrenal (HPA) hormonal responses in adolescent and adult rats [55,57,60,61,62,63,64,65], which is a pattern of effects roughly opposite to that displayed by early-life stress [8,14,15,46,47]. Of special relevance are the findings indicating that, in adolescent rats, H treatment reduces morphine-, cocaine- and amphetamine-induced conditioned place preference, diminishes activity and impulsivity in a familiar open field arena [66] and also has a protective effect on the cortex, HPC and striatum, as evidenced by lower oxidative damage even following repeated cocaine administration [65,67,68]. In a related vein, in adult (PND90) rats, H treatment enduringly reduces locomotor activity under repeated testing (2 h per day, three consecutive testing days), decreases the dopamine response to stress in the NAcc core, and reduces dopamine D3 receptor density in the NAcc shell, which might be responsible for the H-induced reduction of the locomotor-stimulating effects of cocaine [69]. H administered during the first 12 days of life has also been shown to increase the density of pyramidal dendritic spines in vHPC and PFC (at PND35; early adolescence) and dendritic length in PFC (at PND35, and at PND60; late adolescence or young adulthood) in rats, while decreasing dendritic arborization in the NAcc at PND60 [70]. Remarkably, in genetically anxious and stress-sensitive rats, H reduces HPC and AMY volumes in association with very long-lasting anti-anxiety and anti-stress effects [57,62,63].

In sum, H treatment enduringly reduces anxiety and AMY-mediated stress responses, improves attention and HPC-dependent cognitive processes (including cognitive flexibility and working memory, among others), decreases NAcc- and dopamine-mediated responses (such as psychostimulant- and morphine-induced place preference, and others), improves HPC long-term potentiation (LTP), prevents HPC oxidative damage and increases HPC neurogenesis, and promotes increases in spine densities in HPC and PFC. Moreover, many of these effects have been observed during adolescence.

Much like the effects of H, EE in rodents also displays enduring anxiolytic-like effects and reduces AMY- and HPA axis-mediated stress responses, induces attention and cognitive improvements in many (spatial and non-spatial, conditioned and unconditioned) tasks, improves HPC plasticity (e.g., LTP), and increases HPC neurogenesis in adult rodents (see [71] and references therein; reviewed in [54,55]).

Most importantly, EE administered during adolescence in rats has been shown to be able to normalize prepubertal stress (PPS)-induced impairment of adult neuroplasticity (LTP) and neurogenesis in the HPC, to reverse PPS-induced behavioural impairments, and to normalize PPS-linked AMY function and HPC-dependent cognitive alterations (see [71] and references therein).

In a related vein, it is worth highlighting that human MRI studies have evidenced that positive maternal behaviour during early adolescence (11–12 years of age) was associated four years later with attenuated AMY volume and decreased PFC thickness [72]. In a related study, the authors evaluated whether positive maternal behaviour could normalize (or influence) the brain developmental effects of living in a socioeconomically disadvantaged neighbourhood. After over six years of follow-up (the first MRI scan was performed at approximately 12 years of age, and the last scan was done at 19 years of age), positive maternal behaviour was shown to moderate the negative effects of socioeconomic neighbourhood disadvantage on AMY and PFC ([73]). These are very outstanding findings, as they for the first time suggest that some kinds of maternal interactions during late childhood or early adolescence have the potential to influence (and, supposedly, optimize) developmental trajectories of threat- and regulation-related structures (i.e., AMY, PFC). It would be desirable for future studies to establish whether similar juvenile experiences are also capable of shaping the maturational trajectories of the HPC and NAcc, as well as whether adolescent threat- and reward-related behaviour may be influenced by these maternal experiences. In this regard, rat studies on the effects of maternal behaviour have shown enduring positive effects on cognition and anxiety, and on stress-related neuroendocrine processes and neuroplasticity of the HPC and AMY in adult offspring from high licking-grooming mothers (i.e., dams showing high-quality mother care). However, these studies are different from the above human studies in that mother–pup interactions were assessed during the first 10 days postpartum [74,75]. Despite that difference, it is still worth noting that both types of studies demonstrate neuroplasticity changes as a consequence of maternal care. Enhanced maternal care (during the first 10 postnatal days) has also been shown to increase social play in adolescent male rats ([76]). Interestingly, social play has been related to the integrity of the SHPC system, the AMY and the NAcc dopaminergic system [48,53,77,78,79]. Also remarkably, H leads to improved maternal behaviour, in parallel to neural, neuroendocrine and behavioural effects that are in many respects very similar to those observed in offspring from “high maternal care” mothers (for review, see [75]).

In any case, the focus of the present commentary article was to draw attention to the fact that infantile-juvenile enriched and pleasurable experience, such as that provided by H, EE or positive maternal care, has long-lasting positive effects on brain and behaviour during adolescence. The evidence indicates that these early pleasurable experiences are able to shape the adolescent neurodevelopmental trajectories of threat- and reward-related regions such as the AMY, NAcc, HPC and PFC. Importantly, the evidence also shows that such positive experiences are even capable of reversing the deleterious brain developmental effects of previous early (infantile) stress.

It is noteworthy that these four structures are very sensitive to the deleterious effects of early trauma or chronic stress (either administered during the infantile-juvenile period or in adulthood). Thus, this demonstrates that these are very plastic regions, specifically during the juvenile and adolescent periods, and that their plasticity can be influenced by infantile-juvenile experiences in both directions (e.g., [8,14,15,46,47,52,55,56,58,63,71,75] and references therein).

Collectively, the above evidence makes it tempting to suggest that the maturation of the comparator (and regulator) function of the HPC is favoured by some types of early-life pleasurable experiences (such as those mentioned above). This seems to lead to decreased (AMY-mediated) stress- and threat-related processes as well as to reduced (NAcc-mediated) reward-related mesolimbic dopaminergic activity during adolescence. Accordingly, I would suggest that in order to progress in our knowledge on the circuitry underlying the particular adolescent neurobehavioural profiles (or tendencies), we should take into consideration the role of the HPC as a conflict detector and comparator interfaced between the threat avoidance (AMY-related) and reward-driven (VS-related) approach systems (Figure 1) (see, for example, [8,9,10,11,20]).

We need systematic neurobehavioural developmental research (in both rodents and humans) including, or combining: (i) testing situations involving approach–avoidance conflict (rather than tests/tasks focused only on threat (e.g., fear conditioning) or reward separately); (ii) neurobiological manipulations and/or measures (e.g., brain lesions; optogenetic stimulation; brain molecular measures; neuroimaging, etc.) at the level of the key regions of the above “tetradic” circuitry; and (iii) infantile-juvenile enriched experience conditions likely to have positive neurodevelopmental influences on the neural circuitry involved in adolescent behaviour.

## 5. Conclusions

To sum up, to the extent that approach–avoidance conflict in adolescents is a common situation, basic and human neurobiological research focused on the adolescent period should take into account the HPC system because of (i) its regulatory role of AMY, NAcc and PFC function, and (ii) its well-established role as a conflict detector and comparator that regulates whether an approach (e.g., risk taking, sensation/reward seeking) or an avoidance (behavioural inhibition) response will be chosen when facing a conflict situation. This nevertheless implies that the behavioural processes or responses (i.e., the dependent variables) under study in relation to HPC function should include procedures involving goal/response competition (i.e., conflict between incompatible goals or behavioural responses) such as, for instance, between threat- and reward-driven responses, rather than just focusing on one of these processes.

I propose a “tetradic” model, directly derived from J.A. Gray’s neurobiological model [10,11,12,26,27,36], which might complement the “triadic” model [3,6,13,16,17] by integrating the comparator role of the HPC system with that of the other three structures and related circuitry (Figure 1).

As discussed above, it would also be desirable to consider early-life pleasurable enriched experience as a factor (as opposed, or complementary, to early-life adversity factors) that can shed light on neuroplasticity processes (in the HPC, PFC, AMY and VS; and maybe in other regions) of relevance to better understand how the adolescent “brain regional-circuitry function → behavioural output” relationship works, how can it be modulated, and what mechanisms or interactions among those brain regions/circuits may lead to more “balanced” adolescent responses to demands.

One could ask: why put forth efforts to change a neurocircuitry development that appears to be healthy and adaptive for the adolescent? This is an important theoretical issue. The first nuance to point out is that many adolescents may be affected by the deleterious neurobehavioural effects of early-life adversity or, in general, may have maladaptive personality-behavioural profiles that might be shaped by environmental influences. As reviewed here, in some cases in which some kind of vulnerability to juvenile psychopathology or to maladaptive behaviour could be predicted, certain types of (pleasurable) enriched experiences such as those mentioned in this paper (or similar) could help to modulate—and perhaps to optimize—both the neurodevelopment and the behavioural responses of these adolescents. As discussed in the present paper, that contention is strongly supported by basic animal research, and some findings with adolescent humans tend to point in a similar direction.

On the other hand, the present commentary (and the reviewed literature) points out that certain infantile-juvenile enriched experiences have long-lasting “positive” neuroendocrine, neuroplasticity and behavioural effects in rodents, which are already observed at a very early age, and also during adolescence, adulthood and old age. Many of these effects have been observed in the target regions that have been discussed in this article, which in turn are very sensitive to the effects of stress. Hence, early enriched (pleasurable) stimulation treatments constitute a tool with important heuristic potential to improve our understanding of the function of these structures and circuits, as well as their relationship with adolescent behaviour.
